# Increasing Minimum Daily Temperatures Are Associated with Enhanced Pesticide Use in Cultivated Soybean along a Latitudinal Gradient in the Mid-Western United States

**DOI:** 10.1371/journal.pone.0098516

**Published:** 2014-06-11

**Authors:** Lewis H. Ziska

**Affiliations:** Crop Systems and Global Change Laboratory, United States Department of Agriculture, Agricultural Research Service, Beltsville, Maryland, United States of America; US Geological Survey, United States of America

## Abstract

Assessments of climate change and food security often do not consider changes to crop production as a function of altered pest pressures. Evaluation of potential changes may be difficult, in part, because management practices are routinely utilized *in situ* to minimize pest injury. If so, then such practices, should, in theory, also change with climate, although this has never been quantified. Chemical (pesticide) applications remain the primary means of managing pests in industrialized countries. While a wide range of climate variables can influence chemical use, minimum daily temperature (lowest 24 h recorded temperature in a given year) can be associated with the distribution and thermal survival of many agricultural pests in temperate regions. The current study quantifies average pesticide applications since 1999 for commercial soybean grown over a 2100 km North-South latitudinal transect for seven states that varied in minimum daily temperature (1999–2013) from −28.6°C (Minnesota) to −5.1°C (Louisiana). Although soybean yields (per hectare) did not vary by state, total pesticide applications (kg of active ingredient, ai, per hectare) increased from 4.3 to 6.5 over this temperature range. Significant correlations were observed between minimum daily temperatures and kg of ai for all pesticide classes. This suggested that minimum daily temperature could serve as a proxy for pesticide application. Longer term temperature data (1977–2013) indicated greater relative increases in minimum daily temperatures for northern relative to southern states. Using these longer-term trends to determine short-term projections of pesticide use (to 2023) showed a greater comparative increase in herbicide use for soybean in northern; but a greater increase in insecticide and fungicide use for southern states in a warmer climate. Overall, these data suggest that increases in pesticide application rates may be a means to maintain soybean production in response to rising minimum daily temperatures and potential increases in pest pressures.

## Introduction

Considerable research effort has focused on determining the impact of anthropogenic climate change on global agriculture [1]–[4]. Of merited interest in this regard are the physical aspects of climate (e.g. carbon dioxide, temperature, precipitation, extreme weather events) that directly alter crop biology (e.g. growth, phenology, sterility, yields) and the resulting consequences for food security [5]–[9].

However, research efforts related to assessing the agricultural impacts of rising CO_2_ and climate change do not always consider trophic interactions. Overall, changes to the biology and competitive abilities of agricultural pests (insects, pathogens, weeds) relative to potential crop yield losses has not been well quantified [10]–[11]. This is an important omission as the role of pests on constraining crop production is significant and well recognized. For example, weed competition can result in potential crop losses of ∼34% globally, with insect pests and pathogens resulting in additional losses of ∼18 and 16%, respectively [12].

Such omissions may reflect the complex challenges in relating atmospheric CO_2_ and climate variables to potential reductions in crop production related to increased pest pressures. For example, weed growth and fecundity can be directly affected by increasing atmospheric CO_2_ as well as rising temperature; insects and pathogens can also be directly affected by temperature, but indirectly by CO_2_ and/or climate induced changes to their weed hosts [12]–[13]. Overall, while a number of pest studies have been conducted, empirical evidence has been eclectic, although it has been suggested that pest pressures will probably increase with climate change (e.g., [14]).

Yet, even if pest pressures regarding crop production were unequivocal and well-characterized in regard to climate change, it would still be difficult to quantify yield reductions *in situ*. This is because there are strong economic incentives at the field level to manage agro-ecosystems to prevent or minimize pest damage. While management methods vary greatly and may include cultural, mechanical, chemical and biological options, among developed countries, such as Australia and the United States, application of chemicals, usually as pesticides, represents the most widely used method for pest control.

But could quantification of pesticide usage in turn, provide an alternative means to gauge changes in pest pressures associated with changes in climate? Among climate variables, it is generally recognized that in temperate regions, the distribution and survival of agricultural pests is often limited by low winter temperatures; i.e., minimum thermal thresholds) [15]. Rising minimum temperatures associated with anthropogenic climate change could extend the potential geographic range of pest species and/or alter their demographics, although long-term changes in species diversity are unclear [16]–[17]. Climate change assessments have also emphasized that the current and projected increases in global warming are not uniform, and enhanced land-surface temperatures (relative to the global average) are more probable for minimum (Winter) than maximum (Summer) temperatures [18]–[19].

To ascertain if minimum temperatures reflect pesticide usage, regression analysis of the interrelationship between insecticide, fungicide and herbicide application rates and the minimum daily (24 h) observed temperature was conducted on a commercial crop, soybean, grown over a latitudinal transect of seven mid-western states within a humid region. This analysis was performed using a multi-year (1999–2013), multi-state (Minnesota, Wisconsin, Iowa, Missouri, Arkansas, Mississippi and Louisiana) data series obtained from USDA-NASS pesticide usage surveys in conjunction with state based minimum temperature. If significant correlations were observed between minimum temperature and usage for a given class of pesticide, longer trends (1977–2013) in minimum temperature were analyzed and then used to project near-term (decadal, to 2023) changes in pesticide rates by state.

## Results

**Figure 1:**
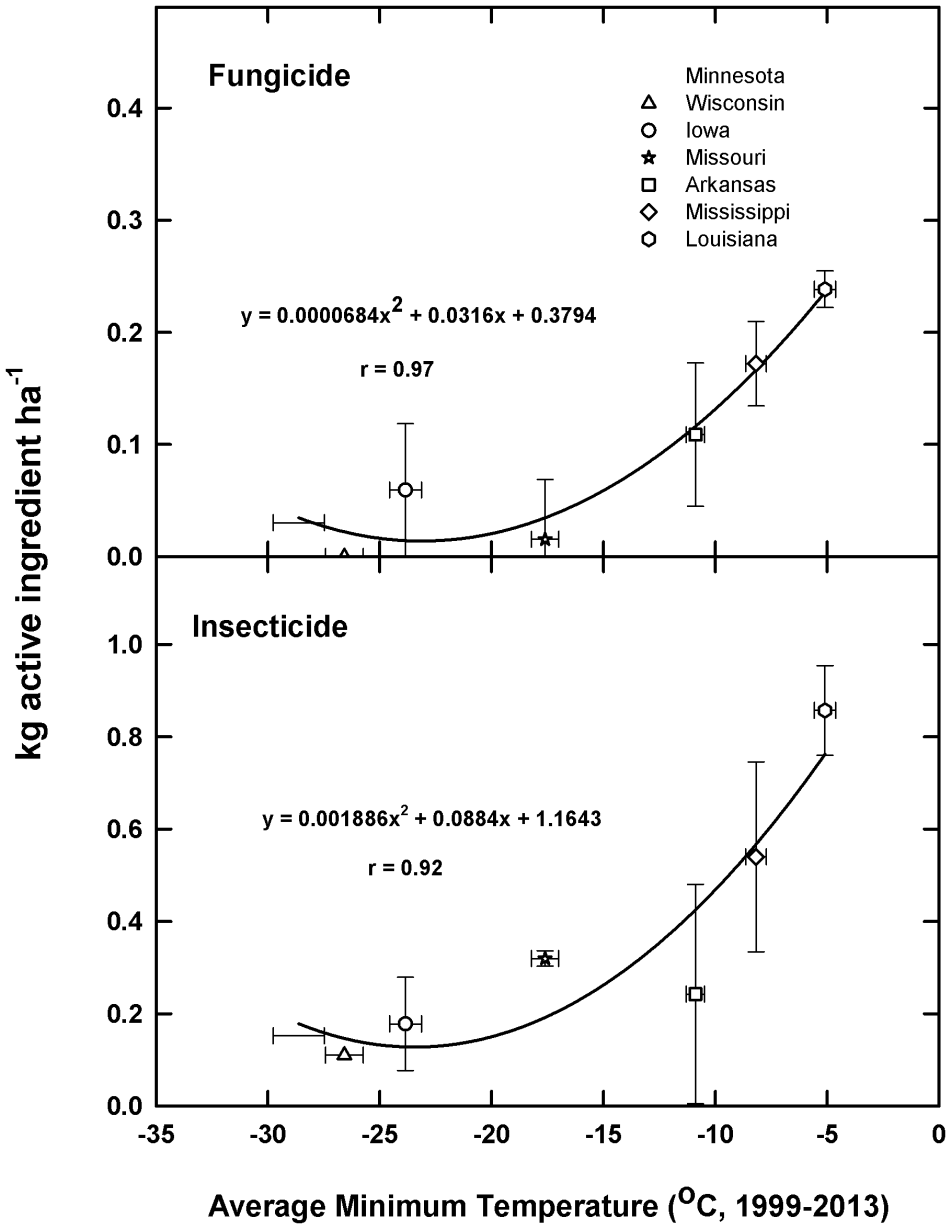
Minimum temperature by state averaged for the period 1999–2013 as a function of insecticide and fungicide usage (kg active ingredient, ai, per hectare) as determined from the National Agricultural Statistical Service survey for the years 1999, 2000, 2001, 2002, 2004, 2006, and 2012) for a north-south transect of seven Midwestern states where commercial soybean is grown. Minimum temperatures were determined as the lowest recorded temperature for a 24“best fit” second order polynomial. Bars are ±SE. See Methods for additional details.

**Figure 2:**
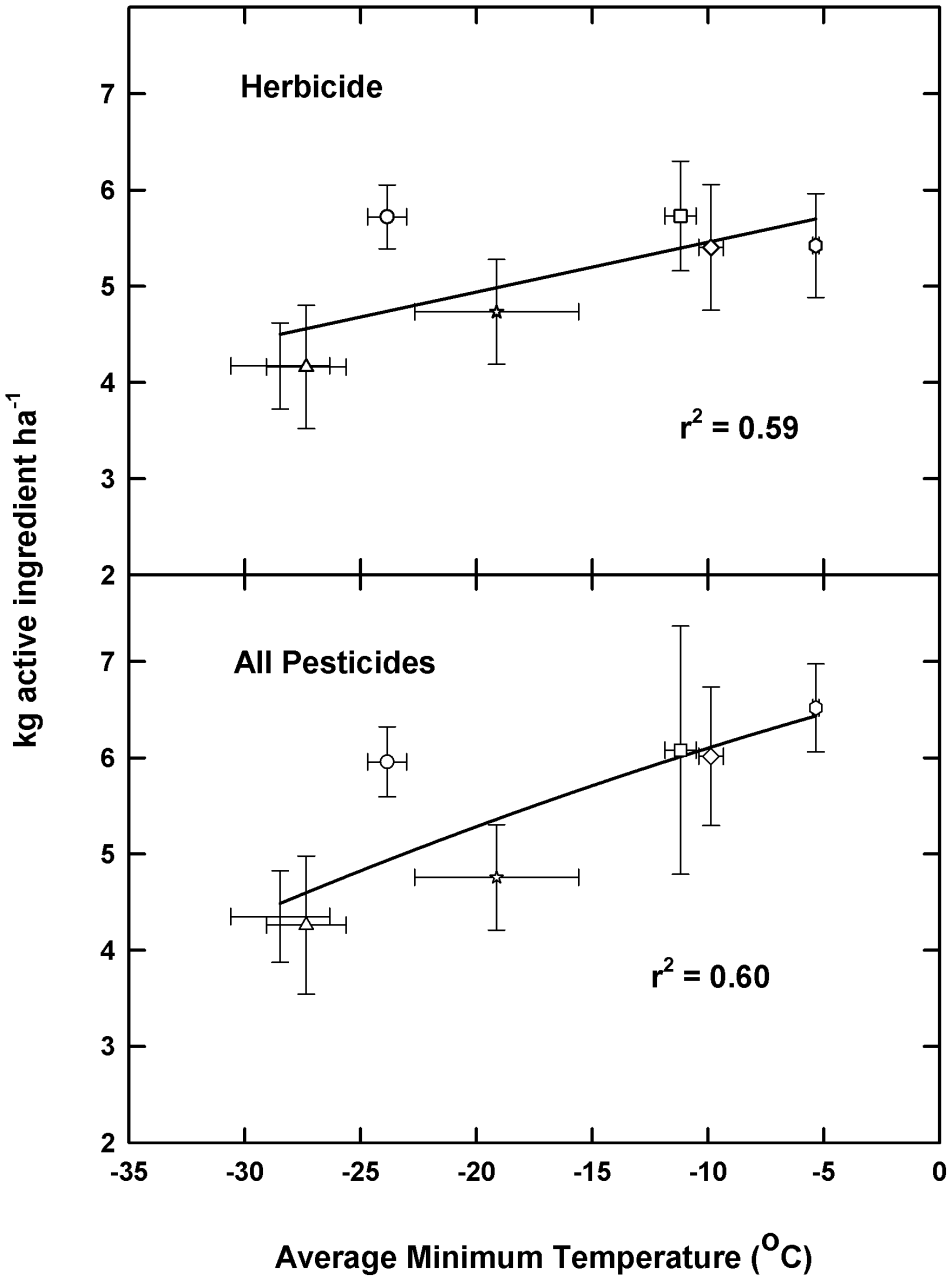
Symbols and regression determined as for figure 1, but for herbicide use and total pesticide (insecticide, fungicide and herbicide) usage for soybean.

For two classes of pesticides, fungicides and insecticides, a second order quadratic function provided the ‘best-fit’ for minimum temperature and amounts of active ingredient applied for the period 1999–2012. There was a significant correlation (r values of 0.97 and 0.92, **,P<0.01) for fungicide and insecticide soybean applications, respectively over the north-south transect (**Figure 1**). A linear function between minimum daily temperature and herbicide application rates was also observed to be positively correlated and significant (r value of 0.84: *,P<0.05) for herbicide use (**Figure 2**). As most pesticide applications are herbicides, a similar relationship (r = 0.84) was observed for total pesticide use (**Figure 2**).

**Figure 3:**
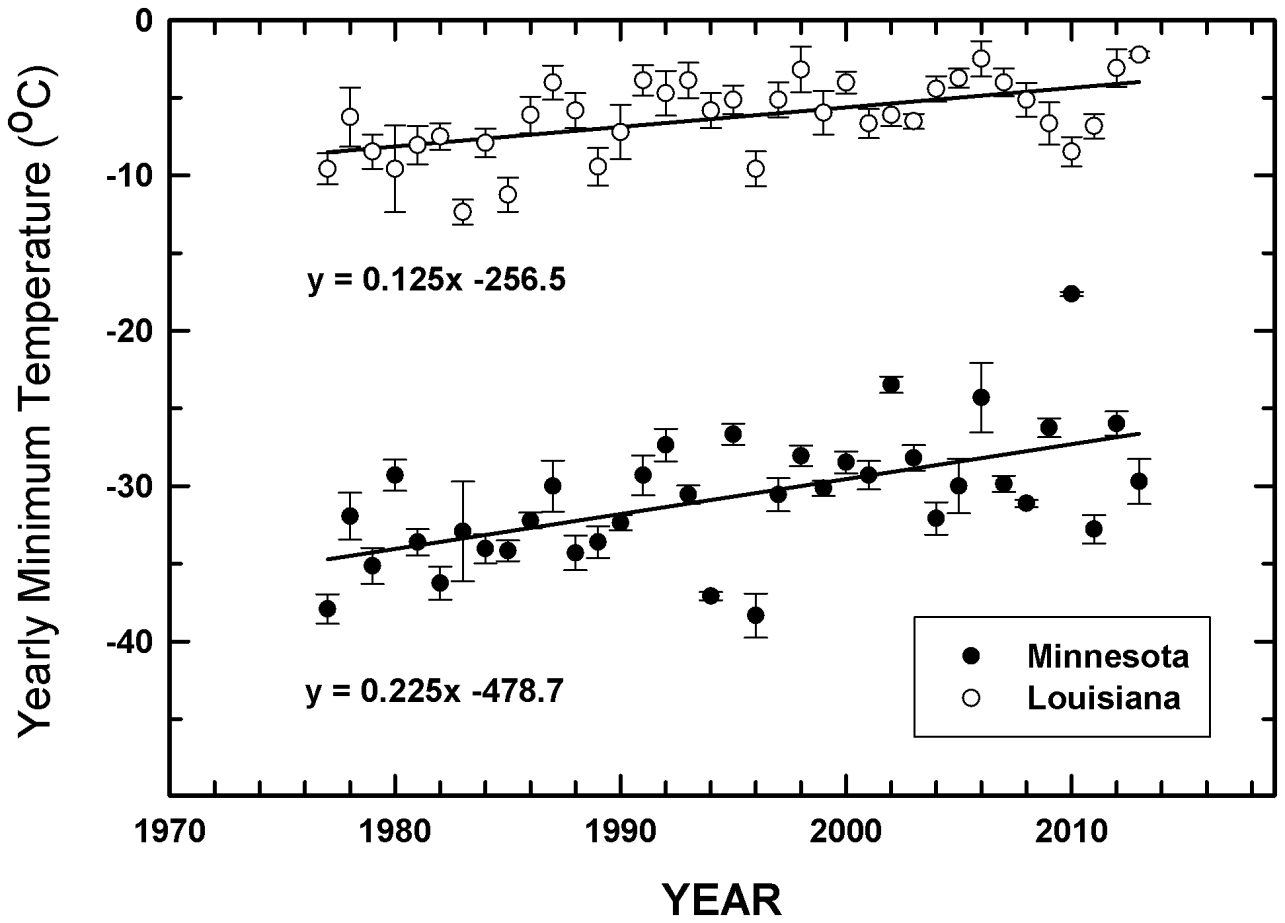
Change in average (±SE) minimum temperature (lowest recorded temperature for a 24 h period during a given calendar year for four locations) for the northernmost and southernmost states (Minnesota, closed circles) and Louisiana (open circles) used in the Midwestern transect. Slope of the regression line indicates the average increase in minimum temperature (°C) per year for each location.

As the correlation between minimum daily temperature and pesticide usage was significant in all cases, states within the transect were examined to quantify longer term temperature changes (1977 through 2013) in order to assess the increase in minimum winter temperature per decade. Overall, these data indicated that the rise in minimum temperatures was a function of latitude, with states such as Minnesota showing a more rapid increase in minimum temperatures than southern states (e.g., Louisiana) (**Table 1** **,** **Figure 3**). However, average soybean yields (2009–2013, MT Ha^−1^) did not significantly vary as a function of the north-south transect (**Table 1**).

**Table 1:** States included in the latitudinal transect.

State	Soybean(Ha *1000)	Soybean(MT Ha^−1^)	T_min_/decade	r value	Insecticide(2023)	Fungicide(2023)	Herbicide(2023)
Minnesota	2,513	2.81	+2.25	0.58,**	0	0	+160.2
Wisconsin	628	2.91	+1.99	0.57,***	0	0	+140.0
Iowa	3,741	3.20	+1.58	0.51,**	+20.3	0	+85.1
Missouri	2,247	2.52	+1.56	0.51,**	+35.6	+12.0	+73.5
Arkansas	1,308	2.62	+1.31	0.54,***	+110.0	+36.5	+70.0
Mississippi	806	2.77	+1.31	0.52,***	+117.7	+39.3	+70.3
Louisiana	449	2.81	+1.25	0.55,***	+170.0	+41.1	+58.6

Soybean area is given as hectares (*1000), average yield was determined from 2009–2013 as metric tons (MT) per hectare, the T_min_ is the increase in minimal daily (24 h) temperature (°C) per decade (determined from 1977–2013). The r value is the correlation coefficient of T_min_ over time, with P<0.05, *; P<0.01, ** and P<.001, ***. The values for insecticide, fungicide and herbicide are determined for 2023 (10 year projection) using the functions for figures 1–3 and are shown as changes in grams of active ingredient (ai) per hectare, with a value of zero indicating no change in application rates over 2013 levels.

The average decadenal increase in minimum daily temperature was used to estimate potential near term (to 2023) changes in pesticides by category. Because a first order regression was the best-fit for herbicide use, increases in minimum temperature result in a relatively greater increase in herbicide applied for northern compared to southern states. In contrast, because the relationship between insecticide/fungicides and minimum temperatures is best described by a second degree polynomial, temperature changes above a critical threshold, (ca −20°C, **Figure 2**) resulted in proportionally larger changes in insecticide/fungicide usage. Hence, for those states with minimum temperatures above this threshold, further increases in temperature result in disproportionally larger projected changes in insecticide and fungicide usage (**Table 1**).

## Discussion

The need to assess the relative impact of pests on agricultural production in a changing climate has long been recognized [20]; however, few quantitative *in situ* assessments are available. An earlier economic analysis [21] indicated increased pesticide costs as a function of increased rainfall and hotter weather during the 1990s. Application of this analysis to projected climate scenarios to 2090 also indicated general increases in average pesticide cost for a range of crop species [21]. However, this study, while useful, did not distinguish between classes of pesticide, or quantify changes in application amounts as a function of a specific climate variable.

Although agriculture is widespread, there are, in fact, only a handful of crops that are grown over a wide range of climates. For the U.S., soybean, corn and wheat fall into this category; however wheat is grown temporally over a latitudinal gradient as spring (e.g. North Dakota) or winter wheat (e.g. Kansas) with significant differences in pest management. In addition, wheat is not grown widely in the southern states. In contrast, corn is often grown in conjunction with soybean, but data on pesticide use for corn in the southern states (e.g. Mississippi, Arkansas, and Louisiana) is currently not available through NASS. Alternatively, the distribution of soybean in this study encompassed a broad range of minimum daily temperatures (i.e., a ∼23°C difference from Minnesota to Louisiana). Such a range would include projections of future surface temperatures associated with anthropogenic climate change, e.g. if minimum daily temperatures in Minnesota became like those of Louisiana [22]; hence soybean was selected for analysis.

There is, as shown here, a significant positive correlation between the range of minimum daily temperatures and pesticide usage for soybean, particularly in regard to fungicides and insecticides. The second order functions for soybean are consistent with insect and fungal biology; i.e., once temperature has reached a critical thermal threshold, it is a significant driver of shifts in insect and pathogen demography [13], [23]. For example, in the UK increasing winter temperatures above a biological minimum have been associated with increased northward migration of aphids in Scotland and increased genotype variation among aphid populations [24]. Similar correlations have also been observed between minimum winter temperatures and the southern pine beetle [25].

Patterson et al. [14] provide an extensive list of temperature thresholds in this regard including associated phenological responses and potential shifts in the expansion of insect ranges. These data indicate that for temperate regions, warming would result in increased winter survival as well as an increase in growth and insect fecundity. Similarly, it has been recognized that pathogen development is affected by warm conditions, with mild winters and warmer weather associated with increased outbreaks of powdery mildew; leaf spot disease, leaf rust and rizomania disease [14]. Treharne [26] in turn, has also suggested that increases in minimum daily temperatures would shift the occurrence of plant disease into cooler regions. The relationship between insecticide and fungicide use observed here for soybean in response to rising minimal temperatures is, overall, consistent with the biology of thermal limits and expected shifts in insect and fungal populations in a warmer climate (e.g., [14], [23], [27]).

For the current study, herbicide applications were a linear function of rising minimum daily temperatures in soybean. In contrast to insect and fungal populations, thermal limits for the occurrence of weeds in soybean were not evident. However, if climate is suitable for crop production, it will also be, de facto, suitable for weed growth.

Although herbicide tolerance in agriculture is increasing [28], for the current study the observed increase in herbicide applications with minimum temperatures did not appear to be related to a greater number of herbicide resistant weeds along the north-south transect (**Table 2**). Nor was there any apparent relationship with photosynthetic pathway (i.e. the number of C_4_ weeds is greater in Minnesota than Louisiana). This suggests no difference in recent CO_2_ increases on latitudinal weed selection between C_3_ and C_4_ weedy species *per se*. However the number of perennial or facultative perennial weed species was significantly greater for the southern soybean locations (e.g. Louisiana, **Table 2**). In weed management, it is generally recognized that perennial weeds can be more competitive and more difficult to control. More competitive because of their ability to quickly regenerate from below-ground structures in the spring and more difficult to control chemically because the elimination of perennial weeds requires the killing of all plant parts (including belowground stems, rhizomes, tubers, etc.) that are capable of producing new shoots [29]. Potential increases in perennial weed establishment with increasing minimum temperatures could, in turn, result in concomitant increases in herbicide usage.

**Table 2:** Top ten lists of most troublesome weed species in soybean for three states along the North-South transect.

State	Common Name	Scientific Name	PhotosyntheticPathway	Growth Habit
*Louisiana*	Morning glory spp.	*Ipomea spp.*	C_3_	Annual or Perennial
	Pigweed spp.	*Amaranthus spp.*	C_4_	Annual or Perennial*
	Browntop millet	*Urochloa ramose*	C_4_	Perennial
	Nutsedge spp.	*Cyperus spp.*	C_4_	Perennial
	Redvine	*Brunnichia ovate*	C_3_	Perennial
	Henbit	*Lamium amplexicaule*	C_3_	Annual*
	Dayflower	*Commelina spp.*	C_3_	Annual or Perennial
	Johnson grass	*Sorghum halapense*	C_4_	Perennial
	Prickly sida	*Sida spinosa*	C_3_	Annual
	Red rice	*Oryza sativa*	C_3_	Annual
*Missouri*	Waterhemp	*Amaranthus rudis*	C_4_	Annual*
	Morning glory	*Ipomea spp.*	C_3_	Annual or Perennial
	Palmer amaranth	*Amaranthus palmeri*	C_4_	Annual*
	Giant ragweed	*Ambrosia trifida*	C_3_	Annual*
	Johnson grass	*Sorghum halapense*	C_4_	Perennial
	Asiatic dayflower	*Commelina communis*	C_3_	Annual
	Horseweed	*Conyza canadensis*	C_3_	Biennial*
	Prickly sida	*Sida spinosa*	C_3_	Annual
	Common ragweed	*Ambrosia artemisiifolia*	C_3_	Annual*
	Eastern black nightshade	*Solanum ptycanthum*	C_3_	Annual or Perennial
*Minnesota*	Lambsquarters	*Chenopodium album*	C_3_	Annual
	Giant foxtail	*Setaria faberii*	C_4_	Annual
	Waterhemp	*Amaranthus rudis*	C_4_	Annual*
	Wooly cupgrass	*Eriochloa villosa*	C_4_	Annual
	Giant ragweed	*Ambrosia trifida*	C_3_	Annual*
	Yellow foxtail	*Setaria viridis*	C_4_	Annual
	Green foxtail	*Setaria lutescens*	C_4_	Annual
	Quackgrass	*Elytrigia repens*	C_3_	Perennial
	Common ragweed	*Ambrosia artemisiifolia*	C_3_	Annual*
	Wild proso millet	*Panicum miliaceum*	C_4_	Annual

Lists are generated by farmer surveys as reported by the Southern Weed Science Society (SWSS) for Louisiana (LA) and Missouri (MO) [37] and the University of Minnesota extension service for Minnesota (MN) (Frank Forcella, USDA-ARS Personal Communication). C_3_/C_4_ refers to photosynthetic pathway.

*Indicates herbicide resistant populations within that state.

The quantitative differences in decadenal increases in minimum temperature reported here (e.g., 2.25 vs. 1.25°C per decade for Minnesota and Louisiana, respectively since 1977) are consistent with the Intergovernmental Panel on Climate Change (IPCC) projections regarding enhanced warming as a function of latitude [19]. Because of the variation in pesticide category by minimal temperature for soybean, near-term projections (2023) suggest a greater relative increase in herbicide use for the upper Midwest, whereas greater insecticide and fungicide use is projected for the southern states for this same period. This may be due, in part, to the southern states having already surpassed the minimum winter temperatures needed to support insect and pathogen populations for soybean in the southern region. However, if minimum temperatures continue to rise, then similar increases in use for all pesticide categories could occur for the upper Midwest.

At present, soybean in the mid-western United States is grown over a ∼2100 km north-south transect that provides a wide range of yearly temperatures. Although such a range of temperatures should, ostensibly, increase pest pressures and subsequent crop loss, no production losses are evident in average yield per hectare (e.g., average yields in Minnesota and Wisconsin do not differ from those of Mississippi and Louisiana). However, insecticide, fungicide and herbicide use increase significantly in conjunction with minimum daily temperatures along the latitudinal transect. Assuming that prophylactic use is not widespread in any one region, this increase should reflect increased pest pressures *per se*.

It should be emphasized however, that the biological basis for rising minimum temperatures and changes in pesticide usage are likely to be complex. While there are empirical correlations between population demographics and temperature, temperature by itself does not reflect the complexity of pest population causations in agriculture. For example, pest-pest interactions, insects as disease vectors, temporal asynchrony between insects and host plants, soil management and herbicide efficacy (e.g. precipitation, windspeed), *inter alia* [10], [23], [30] will also need to be considered. Rising levels of carbon dioxide are also likely to directly alter secondary plant chemistry and alter plant-arthropod interactions depending on plant species and insect group [31]. In addition, the current analysis assumes future monotonic poleward shifts in minimum temperatures influencing pesticide usage; whereas an increase in extreme climatic events may be the norm [32].

However, the ability to include realistic impacts of agricultural pests in future assessments of climate change and food security is imperative. While there are numerous challenges remaining before the impacts of anthropogenic climate change on pest pressures and crop yields can be completely quantified, the current study suggests that farm management, through the increased use of chemical applications, may negate pest pressures on crop production that are incurred as a result of changing climate variables such as temperature. As such, pest-induced reductions in agricultural yield of soybean associated with climate change may be difficult to quantify where pesticides are widely used. At present, while the current analysis suggests that minimum daily temperatures could be used as a proxy to understand climate change impacts on pesticide use in soybean; critical information regarding the environmental and economic consequences of such impacts will require further study.

## Materials and Methods

### Soybean

For all seven states examined along the north-south transect, soybean is considered a major crop (>300,000 ha planted) [34]. Genetically modified (gmo) soybean (e.g., Round-Up Ready^tm^) was introduced in 1995–1996, mainly as a means for blanket control of post-emergent weeds. Following its introduction it was quickly adopted by growers, with gmo soybean acreage >50% by 1999 [33] (**Table 3**). There is no evidence of different rates of adoption of gmo soybean over the period 1999–2012. For example, by 2001, the amount of soybean area plant to gmo was 63% for both Minnesota and Mississippi (**Table 3**). Since 2005, approximately 90% of all soybeans grown in the U.S. have been herbicide resistant gmo (**Table 3**).

**Table 3:** Genetically engineered soybean varieties for herbicide tolerance for states included in the north-south transect.

STATE	2000	2001	2002	2003	2004	2005	2006	2007	2008	2009	2010	2011	2012
Minnesota	46	63	71	79	82	83	88	92	91	92	93	95	91
Wisconsin	51	63	78	84	82	84	85	88	90	85	88	91	92
Iowa	59	73	75	84	89	91	91	94	95	94	96	97	93
Missouri	62	69	72	83	87	89	93	91	92	89	94	91	91
Arkansas	43	60	68	84	92	92	92	92	94	94	96	95	94
Mississippi	48	63	80	89	93	96	96	96	97	94	98	98	95
U.S.	54	68	75	81	85	87	89	91	92	91	93	94	93

Values are the percent of all soybeans planted. Data are available at:

www.usda.mannlib.cornell.edu/usda/nass/Acre/2010s/2011/Acre-06-30-2011.pdf#page=27.

### Pesticide Use Rates

State level pesticide usage rate are available for 1999, 2000, 2001, 2002, 2004, 2006 and 2012 from the USDA National Agricultural Statistical Service Survey (NASS) data online (www.quickstats.nass.usda.gov/#5072A3CD-64E7-384A-8FF0-DDC931164C3B) [34] These data include chemical usage by compound for insecticides, fungicides and herbicides for soybean for the states included in the north-south transect. Although data are also available from 1991 through 1998 from this same source, the rapid rates of gmo adoption for herbicide resistant soybean, and the shift in herbicide usage during this time period was thought to, potentially, obfuscate any potential changes related to climate variables such as temperature. This is because application rates often vary by herbicide mode of action; hence, differential herbicide use would result in different amounts of active ingredient (ai) being applied. However, the large-scale shift to gmo soybean provided greater uniformity in herbicide type over a large regional area (**Table 3**). This is an important consideration as herbicides represent the largest component of total pesticide use (e.g. **Figure 2**). However, the temporal range of yearly averages (1999–2013) was considered insufficiently long to evaluate any potential increase in minimum daily temperatures associated with anthropogenic surface warming and pesticide usage (relative to year to year variability) for any one state.

### Temperature Data

A software program developed by Texas A&M University [35] was used to identify at least four weather stations located within soybean growing regions of a given state used in the transect. Information on the lowest recorded (24 h) minimum temperature for a given year was determined and averaged for all weather stations within a state for the 1999–2013 period. These same stations and data sets were used to generate minimum daily temperature over a longer time period, from 1977 (i.e. the start of the rapid increase in global land-ocean temperature index) through 2013 [36].

### Statistical Analysis

In examining pesticide use it is important to determine whether there may be fixed or random regional effects within the data. For example, are there state specific factors beyond temperature that would result in systematic shifts in pesticide usage? (e.g. one state using irrigation while another state does not, change in rotation rates, etc.) This analysis has been previously done by Chen and McCarl [21] who found with 99% confidence that a random state effect existed for pesticide use in corn, potatoes, soybeans and wheat [21].

A step-wise regression program (ver. 10.0 Statview, Cary NC, USA) was used to determine the best fit regression line for average minimum daily temperature and pesticide application rate (insecticides, fungicides and herbicides) for all states within the latitudinal transect. If the correlation coefficient was determined to be significant for a given pesticide category (e.g. insecticides); then a longer term minimum daily temperature data set (1977–2013) was generated for each state to quantify increases in minimum temperature by decade. The increase in minimum daily temperature by decade was then used to project potential short-term (2014–2023) pesticide use (by category and state) using the quadratic or linear functions provided in figures 1 and 2.
